# 

**Published:** 1898-05-28

**Authors:** 


					|cX THE HOSPITAL. "The standard of highest purity."? Lancet. May 28th, 1898.
CADBURY'S ^ WM fea CADBURY'S
COGOA r.? jri M-7 COCOA
ABSOLUTELY PURE. Wk NO DRUGS OR ALKALIES USED.
WM7?H HOSPl.TAL
No. 609. Vol. XXIV. [J3S51S3 Sattodat,
May 28th, 1898.
[SuhBcriptionTermB.-j pRICE TWOPENCT,

				

